# Visceral adiposity index is strongly associated with hyperuricemia independently of metabolic health and obesity phenotypes

**DOI:** 10.1038/s41598-017-09455-z

**Published:** 2017-08-18

**Authors:** Huimin Dong, Yang Xu, Xiuzhi Zhang, Simiao Tian

**Affiliations:** 1 Department of Clinical Nutrition and Metabolism, Affiliated Zhongshan Hospital of Dalian University, NO. 6 Jiefang Street Zhongshan District, Dalian, Liaoning Province 116001 People’s Republic of China; 2 Department of Scientific Research Project, Affiliated Zhongshan Hospital of Dalian University, NO. 6 Jiefang Street Zhongshan District, Dalian, Liaoning Province 116001 People’s Republic of China

## Abstract

Visceral adiposity index (VAI) is a novel sex-specific index for visceral adipose function; however the association between VAI and hyperuricemia in China is unknown. We aimed to investigate this association, also whether it was independent of metabolic health and obesity phenotypes. 7632 adult subjects from the China Health and Nutrition Survey 2009 were retained. Subjects were categorized into four obesity phenotypes based on a cross-classification of BMI and metabolic health status by two representative criteria. VAI was the best predictors for hyperuricemia irrespective of obesity phenotypes, with area under curve (AUC) ranging 0.665–0.719. The odd ratio (OR) for hyperuricemia in the highest quartile of the VAI were 6.93 (95% CI 5.79–8.29) after adjusting for age and gender. Following further adjustments for metabolic obesity phenotypes and lifestyle confounders, the ORs were 4.88 (3.92–6.09) and 5.65 (4.68–6.82) according to these two criteria, respectively. A similar significant pattern was still found even after adjustment for blood pressure and other cardiovascular risks. Within each metabolic obesity phenotype, the significant association between VAI and hyperuricemia was consistently evident. In conclusion, the association of the VAI with hyperuricemia was significant, especially this association was independent of metabolic health and obesity phenotypes in the Chinese population.

## Introduction

Hyperuricemia has emerged as a major public health concern because of its increasing prevalence and significant impact on various clinical disorders^1–5^. Recent epidemiological investigations have estimated the prevalence of hyperuricemia to be 8.4–25% in the general Chinese population^6^, 25.8% in Japan^7^ and 11.9–16.6% in Western populations^8–10^. Also it is estimated that 21% of the US general population suffer from hyperuricemia^11^. Hyperuricemia has been shown to be an important independent factor that increases the risks of morbidity and mortality associated with many diseases, including hypertension^3^, diabetes mellitus^12^, stroke^13, 14^, dyslipidemia^15^, chronic kidney disease^16^, cardiovascular events, and heart failure^17, 18^. It also plays an important role in contributing to the development of gout, which can impair patients’ quality of life^19, 20^. Accordingly, early diagnosis of subjects with a high risk of hyperuricemia, and preventative intervention, are of special importance in medical practice.

Obesity is recognized as the main cause of a great number of chronic diseases, and also is an established risk factor for development of hyperuricemia. Excess body fat and/or obesity may be linked to excessive serum uric production and poor serum uric excretion, which lead to impaired uric acid (UA) metabolism, or even particularly hyperuricemia^21^. Ishizaka *et al*. found that obesity is closely related to high serum UA level particularly among postmenopausal women^22^. Tsushima *et al*. summarized that adipose tissue could increase UA secretion in obese subjects, which may contributed to overproduction of UA and cause hyperuricemia as a consequence^23^. However, obese people may vary in their body fat distribution and disease risk, for instance, metabolically healthy obese (MHO), the novel obesity phenotype characterised by favourable cardiometabolic profiles whilst being obese, has been showed to be a benign condition, not associated with an increased risk of cardiovascular disease (CVD) morbidity and mortality compared to normal-weight individuals^24^, and some even reported a protective effect of obesity for all-cause mortality if accompanied by a healthy metabolism^25^.

In many studies, obesity was frequently estimated or reflected by anthropometric indices, such as body mass index (BMI), waist circumference (WC) and waist-to-height ratio (WHtR). However, these indices provide limited information on fat distribution^26, 27^, and more importantly, are unable to distinguish between subcutaneous fat and visceral fat masses^28, 29^. It is noted that the accumulation of visceral adipose tissue, as opposed to subcutaneous fat, contributes more to develop UA metabolism disorders^30^. Accurate measurement of visceral fat masses can be obtained from magnetic resonance imaging (MRI) and computed tomography (CT), which are considered to be the gold standards for the quantitative detection of obesity^31, 32^. Nevertheless, these two methods are inconvenient and expensive, and are therefore unsuitable for large-scale epidemiological studies. In 2010, Amato *et al*. developed a novel visceral adiposity index (VAI) on the basis of anthropometric (WC, BMI) and metabolic (triglyceride (TG) and high density lipoprotein cholesterol (HDL-C)) variables, to express both visceral fat distribution and adipose tissue dysfunction^33^. That study, conducted in a Caucasian population, claimed that the VAI is closely correlated with MRI-measured body visceral adiposity^33^, and suggested that it could be useful surrogate maker for visceral adiposity in obesity-related cardiometabolic risk study^34^. Since then, several studies have investigated the relationship between visceral adiposity, estimated by VAI, and various health-related outcomes, and their compelling result emphasized that VAI was significantly associated with metabolic syndrome^35^, arterial stiffness^36^, pre-hypertension and hypertension^37^, pre-diabetes and type 2 diabetes^38, 39^, and mortality^40^ in different ethnic populations.

To date, as far as we know, no previous studies have reported the relationship between visceral adiposity tissue estimated by VAI and hyperuricemia in the Chinese population, and whether the degree of the relationship varies according to different metabolic health and obesity phenotypes; therefore, we aimed to investigate the relationship between visceral adiposity estimated by VAI and hyperuricemia among MHO and other metabolic obesity phenotypes in China, and to compare the risk predictive ability for hyperuricemia between VAI and other obesity indices including BMI, WC, WHtR, and body adiposity index (BAI).

## Methods

### Study population

This study used data from the 2009 wave of the China Health and Nutrition Survey (CHNS) for our analysis. The CHNS is a large-scale longitudinal, household-based ongoing survey designed to examine the effects of the health and nutrition across a set of large provinces with a range of socio-economic conditions. A comprehensive description of the survey and its sampling procedures has been published elsewhere^41^. In brief, starting in 1989, this survey used a multistage, random cluster process to select households from 9 of the 31 mainland provinces. Within these households, original and new household members were longitudinally assessed. Fasting blood samples from participants aged ≥7 years were collected for the first time in 2009. The survey protocol was conducted according to the Declaration of Helsinki, and this survey protocols, instruments, and the process for obtaining informed consent were approved by the institutional review committees of the University of North Carolina at Chapel Hill, the National Institute of Nutrition and Food Safety, Chinese Center for Disease Control and Prevention, and the China-Japan Friendship Hospital, Ministry of Health (R01-HD30880, DK056350, and R01-HD38700). Each survey participant provided written informed consent. All methods were performed in accordance with the relevant guidelines and regulations.

Among 11929 participants in the 2009 wave of the CHNS, the present study comprised 3501 men and 4131 women aged 18–85 years, for whom anthropometric measures and fasting blood sample information were available.

### Anthropometric and biochemical measurements

Anthropometry was measured following standardized procedures by well-trained examiners. Body weight and height were taken with participants barefoot and in light clothing, and measured to the nearest 0.1 kg and 0.1 cm, respectively. WC was measured with an inelastic tape to the nearest 0.1 cm at a midpoint between the bottom of the rib cage and the top of the iliac crest, following exhalation. Hip circumference was measured over thin clothing at the point of the maximum circumference of the buttocks. Both circumferences were measured to the nearest 0.1 cm. BMI was calculated as weight (kg) divided by the square of height (meters). WHtR was calculated as WC (cm) divided by height (cm). BAI was calculated using the formula^42^: ${\rm{BAI}}=(\frac{{\rm{Hip}}}{{{\rm{Height}}}^{1.5}})-18$. Systolic and diastolic blood pressures (SBP/DBP) were measured on the right arm, using mercury sphygmomanometers. Measurements were collected in triplicate with intervals of 10 minutes’ seated rest, and their means were used in analyses.

Following an overnight fast, blood was collected by venipuncture and tested immediately for glucose and hemoglobinA1c (HbA1c). Plasma and serum samples were then frozen, and stored at −86 degree for later laboratory analysis. All blood samples were analyzed in a national central lab in Beijing with strict quality control. Serum levels of fasting plasma glucose (FPG), total cholesterol (TC), HDL-C, TG, and UA were measured by a biochemical autoanalyzer. Details of laboratory analysis were reported in “CHNS, Manual for Specimen Collection and Processing” (http://www.cpc.unc.edu/projects/china/data/datasets/Blood%20Collection%20Protocol_English.pdf) and “A list of biomarkers and methods used to measure them” (http://www.cpc.unc.edu/projects/china/data/datasets/Biomarker_Methods.pdf). Homeostasis model assessment of insulin resistance (HOMA-IR) was calculated by the formula: HOMA-IR = fasting insulin (micro-international units per millilitre) × FPG(millimoles per litre)/22.5. VAI was calculated using the following formulas, as proposed by Amato *et al*.^33^: Males: ${\rm{VAI}}=\frac{{\rm{WC}}}{39.68+(1.88\times {\rm{BMI}})}\times (\frac{{\rm{TG}}}{1.03})\times (\frac{1.31}{{\rm{HDL}}-{\rm{C}}})$; Females: ${\rm{VAI}}=\frac{{\rm{WC}}}{36.58+(1.89\times {\rm{BMI}})}\times (\frac{{\rm{TG}}}{0.81})$ $\times (\frac{1.52}{{\rm{HDL}}-{\rm{C}}}).$

### Definitions of hyperuricemia, and metabolic health and obesity phenotypes

In our present study, hyperuricemia was defined as serum UA ≥420 *μ*mol/L in men and ≥360 *μ*mol/L in women^43^. The BMI was used to define non-obese and obese status, using a cut-off point of 25 kg/m^2^, and this BMI threshold is based on the definition advocated by Western Pacific Regional Office of WHO (WPRO) for obesity in adult Asians^44, 45^. We adopted two published criteria to define metabolically unhealthy status: (1) the Adult Treatment Panel-III (ATP-III) definition of metabolic syndrome^46^, which is having more than two of the following metabolic abnormalities: SBP/DBP ≥130/85 mmHg or use of antihypertensive drugs, TG ≥1.7 mmol/l or use of lipid-lowering drugs, FPG ≥5.6 mmol/l or use of medications for diabetes, HDL-C ≥1.0/1.3 mmol/l for men/women. The WC criterion was not used because of its colinearity with BMI. (2) The HOMA index^47^, where HOMA-IR is in the upper quartile of the HOMA index. According to the cross-classification of BMI categories and metabolic health status, study participants were categorized into 1 of the 4 following phenotypes: metabolically healthy non-obese (MHNO); metabolically unhealthy non-obese (MUNO); MHO; and metabolically unhealthy obese (MUO).

### Statistical analyses

The characteristics of the study population were presented as medians (interquartile range) for continuous variables, or as percentages for categorical variables. Comparison of the four metabolic health and obesity phenotypes were conducted using one-way analysis of variance (ANOVA) tests for continuous variables, and chi-square tests for categorical variables. Receiver-operating characteristic (ROC) analyses were performed to examine diagnostic ability of obesity indices for hyperuricemia. The area under the receiver operating characteristics curve (AUC) and 95% confidence intervals (CIs) were computed to compare the discriminative power of VAI and other adiposity-based measures of hyperuricemia risk. The association between hyperuricemia and VAI was tested using multiple logistic regression models, with odds ratios (ORs) and 95% CIs calculated. In the first set of analyses, we used the first quartile of VAI as the reference. The ORs and their 95% CIs for the presence of hyperuricemia were adjusted sequentially for age and sex (model 1), then further adjusted for urban/rural resident, smoking status, alcohol status and metabolic health-obesity phenotypes (model 2), and finally adjusted for white blood cell, TC, BP, FPG and hs-CRP variables (model 3). We then conducted a second set of analyses, stratified by metabolic health-obesity phenotype, to allow us to examine the association of hyperuricemia risk with VAI in each stratum of obesity phenotype using model 3. A sensitivity analysis was performed including WC criterion in ATP-III definition of metabolic syndrome without regard for its collinearity with BMI, and metabolically unhealthy status is defined if having ≥3 metabolic abnormalities. As no universal definition of MHO, for a supplemental analysis, we adopted a new criteria based on VAI to define metabolically unhealthy status, and the full statistical analysis was replicated when this novel criteria was used. According to Du *et al*.^48^, individuals with a lower VAI value can be considered having metabolically healthy status (i.e. being “metabolically healthy” was defined as VAI <1.59, and “metabolically unhealthy” as VAI ≥1.59). All statistical analyses were conducted with R version 3.2.2 software (R Foundation for Statistical Computing, Vienna, Austria)^49^, and *P*-value < 0.05 was considered statistically significant.

### Data availability

The datasets generated analysed during the current study are publicly available in the website: http://www.cpc.unc.edu/projects/china/data.

## Results

### Characteristics of the sample population

The characteristics of the study population, according to the ATP-III criteria-based metabolic obesity phenotypes, are presented in Table 1. Of the 7632 subjects, 3780 (49.5%), 1673 (21.9%), 892 (11.7%) and 1287 (16.9%) subjects were classified into the MHNO, MUNO, MHO and MUO groups, respectively. Compared with the MHNO phenotype, MHO subjects were more likely to be female, older, non-smokers and non-drinkers, and to exhibit a less favourable risk profile, such as elevated BP, FPG, TC, and LDL-C levels. Another set of biomarkers, including TG, BP, FPG, UA and HOMA-IR, were lower in MHO than in MUO subjects. In addition, compared with MUNO individuals, MHO individuals were less likely to be current smokers and drinkers, had had a less insulin-resistant profiles characterized by lower levels of BP, TG, FBG and HOMA-IR, and higher levels of HDL-C, despite having higher BMI, WC and BAI (Table 1).

**Table 1:** Characteristics of subjects according to obesity status (defined by body mass index) and metabolic health status defined by ATP-III criteria.

	Non-obese		Obese		*P* value
	Metabolically healthy (MHNO) (n = 3780)	Metabolically unhealthy (MUNO) (n = 1673)	Metabolically healthy (MHO) (n = 892)	Metabolically unhealthy (MUO) (n = 1287)	
Age, year	46.5 (36.3–58.5)	55 (44.7–64.8)	49 (40.2–58.2)	53.7 (44.4–62.3)	<0.001
Sex (female), n (%)	2168 (57.4%)	765 (45.7%)	575 (64.5%)	623 (48.4%)	<0.001
Smoker, n (%)	1107 (29.3%)	638 (38.1%)	181 (20.3%)	409 (31.8%)	<0.001
Alcohol drinker, n (%)	1175 (31.1%)	579 (34.6%)	252 (28.3%)	466 (36.2%)	<0.001
Urban resident, n (%)	2618 (69.3%)	1130 (67.5%)	614 (68.8%)	855 (66.4%)	0.2377
BMI, kg/m^2^	21.4 (19.8–22.9)	22.6 (21.1–23.8)	26.5 (25.7–27.8)	27.3 (26–29)	<0.001
WC, cm	77 (71–82.4)	81.8 (76–87)	89.8 (84.5–94.9)	93 (88–98.5)	<0.001
WHtR	0.5 (0.4–0.5)	0.5 (0.5–0.5)	0.6 (0.5–0.6)	0.6 (0.5–0.6)	<0.001
VAI	1.2 (0.8–1.7)	3.1 (2–4.5)	1.5 (1.1–2.1)	3.5 (2.4–5.3)	<0.001
BAI	26.7 (24.2–29.4)	27.2 (24.7–30)	31.8 (28.7–34.4)	31.2 (28.4–34.6)	<0.001
HDL-C, mmol/l	1.5 (1.4–1.8)	1.2 (1.1–1.4)	1.4 (1.3–1.7)	1.1 (1–1.3)	<0.001
LDL-C, mmol/l	2.8 (2.3–3.4)	3 (2.3–3.6)	3.1 (2.6–3.7)	3.1 (2.6–3.8)	<0.001
DBP, mm Hg	76.7 (70–80.7)	82 (78–90)	80 (75.3–84)	86.7 (80–92)	<0.001
SBP, mm Hg	117.3 (108.7–123.3)	130 (119.3–140.7)	120.5 (112–130)	130.7 (120.7–146)	<0.001
FPG, mmol/l	4.9 (4.5–5.2)	5.6 (5–6)	5 (4.7–5.3)	5.5 (5–6.1)	<0.001
TC, mmol/l	4.6 (4–5.2)	4.8 (4.2–5.5)	4.9 (4.3–5.5)	5 (4.5–5.7)	<0.001
TG, mmol/l	1 (0.7–1.3)	1.9 (1.3–2.6)	1.1 (0.8–1.4)	2.2 (1.6–2.9)	<0.001
UA, mmol/l	266 (219–324)	319 (262–382)	278 (230–333)	342 (287–407)	<0.001
HOMA-IR	1.9 (1.4–2.6)	2.8 (1.9–4.3)	2.5 (1.7–3.6)	3.5 (2.4–5.8)	<0.001
hsCRP	1 (0–2)	1 (1–3)	1 (1–3)	2 (1–4)	<0.001
HbA1c, %	5.4 (5.1–5.7)	5.5 (5.2–5.9)	5.5 (5.3–5.8)	5.7 (5.4–6.1)	<0.001
Diabetes, n (%)	33 (0.9%)	155 (9.3%)	9 (1.0%)	135 (10.5%)	<0.001
Dyslipidemia, n (%)	497 (13.1%)	890 (53.2%)	178 (20.0%)	850 (66.0%)	<0.001
Hypertension, n (%)	525 (13.9%)	733 (43.8%)	210 (23.5%)	703 (54.6%)	<0.001

Data are n (%) or median (interquartile range). Abbreviations: ATP-III, the Adult Treatment Panel-III; BMI, body mass index; WC, waist circumference; WHtR, waist-to-height ratio; VAI, visceral adiposity index; BAI, body adiposity index; HDL-C, high-density lipoprotein cholesterol; LDL-C, low-density lipoprotein cholesterol; DBP, diastolic blood pressure; SBP, systolic blood pressure; FPG, fasting plasma glucose; TC, total cholesterol; TG, triglycerides; UA, uric acid; HOMA-IR, homoeostatic model assessment of insulin resistance; hsCRP, high-sensitivity C-reactive protein.

**Figure 1:**
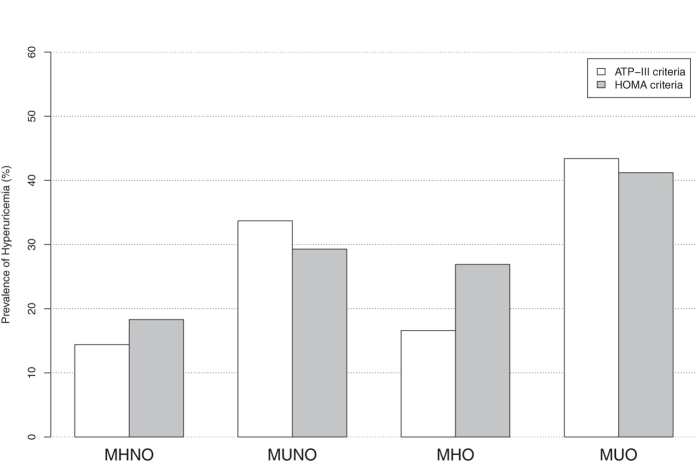
Prevalence of hyperuricemia according to metabolic health and obesity status (metabolic obesity phenotypes). The metabolic health status was defined by ATP-III and HOMA criteria, respectively; the obesity status was defined by body mass index. *Abbreviations*: ATP-III, the Adult Treatment Panel-III; HOMA, homeostasis model assessment of insulin resistance; MHNO, metabolically healthy non-obese; MUNO, metabolically unhealthy non-obese; MHO, metabolically healthy obese; and MUO, metabolically unhealthy obese.

The cardiovascular risk factors were more prevalent in metabolically unhealthy groups than in healthy groups, regardless of BMI level. The prevalence of hypertension, diabetes and dyslipidemia ranged from 54.6%, 10.5% and 66.0% in MUO subjects to 13.9%, 0.9% and 13.1% in MHNO subjects, respectively. The prevalence of hyperuricemia increased with weight for both metabolically healthy and metabolically unhealthy subjects. The highest prevalence of hyperuricemia was consistently found in MUO phenotypes regardless of definitions, being 43.4% and 41.2% according to the ATP-III and HOMA criteria, respectively. The MUNO phenotype revealed a higher prevalence of hypertension than did MHO subjects under both criteria, with nearly one-third of MUNO subjects suffering from hyperuricemia (Fig. 1). In addition, the characteristics of the study participants and the prevalence of hyperuricemia in different VAI criteria-based metabolic obesity phenotypes are shown in Supplementary Table 3 and Supplementary Figure 1. The descending order of hyperuricemia prevalence in VAI criteria-based obesity phenotypes was in agreement with ATP-III and HOMA criteria, and the prevalence was 38.4% in MUO subjects, 30.4% in MUNO subjects, 16.3% in MHO subjects and 12.1% in MHNO subjects, respectively (Supplementary Figure 1).

### ROC analysis

We assessed the potential power of VAI to discriminate between the presence or absence of hyperuricemia and compared it with that of BMI, WC, WHtR and BAI, using ROC analysis (Tables 2 and 3). For ATP-III criteria-based metabolic obesity phenotypes, VAI and BAI were the two best indices with the strongest discriminative ability for hyperurcemia in the four obesity phenotypes. The VAI exhibited the highest diagnostic accuracy for hyperurciemia in MUNO and MUO phenotypes, with AUC values of 0.672 (95% CI 0.644–0.699) and 0.649 (95% CI 0.619–0.679), respectively; whereas BAI showed better discriminative power in MHNO and MHO phenotypes, with corresponding AUC of 0.638 (95% CI 0.613–0.662) and 0.697 (95% CI 0.651–0.743). A sensitivity analysis, including abdominal obesity (WC criterion) into ATP-III definition of metabolic syndrome, showed highly consistent results on AUC values (Supplementary Table 1). Similar pattern was noted that the VAI demonstrated better predictive abilities in identifying hyperurciemia in MUNO and MUO phenotypes, with corresponding AUC values of 0.668 (95% CI 0.636–0.7) and 0.645 (95% CI 0.614–0.675), respectively. In term of HOMA criteria-based metabolic obesity phenotypes, VAI consistently demonstrated the highest discriminative power for hyperuricemia, irrespective of obesity phenotypes. Its AUC values ranged from 0.665 (95% CI 0.628–0.703) in the MUO phenotype to 0.719 (95% CI 0.684–0.755) in the MUNO phenotype. Compared with VAI, BMI and WHtR were the weakest surrogates for the obesity index in term of identifying hyperuricemia, with the AUC range of 0.512–0.593 and 0.509–0.553 depending on the criteria used for metabolic obesity phenotypes. In the remaining analysis, we focused on the associations of hyperuricemia with VAI within different metabolic obesity phenotypes, since the VAI had the best discriminative power for hyperuricemia.

**Table 2:** Areas under the receiver operating characteristic curve (AUC) for identifying hyperuricemia risk with various adiposity measures within four metabolic health and obesity phenotypes defined by ATP-III criteria.

Obesity phenotype		AUC	Threshold	Specificity	Sensitivity	Accuracy
MHNO	BMI	0.515(0.489,0.542)	23.12	78.22	25.78	70.69
	WC	0.585(0.56,0.611)	76.25	50.26	62.25	51.98
	WHtR	0.518(0.492,0.544)	0.47	43.37	62.25	46.08
	VAI	0.62(0.594,0.647)	1.5	70.03	48.8	66.98
	BAI	**0.638(0.613,0.662)**	27.03	50.91	72.19	53.97
MUNO	BMI	0.563(0.534,0.592)	22.98	60.5	50.89	57.26
	WC	0.59(0.562,0.619)	83.25	62.94	51.6	59.12
	WHtR	0.515(0.486,0.544)	0.49	36.07	69.15	47.22
	VAI	**0.672(0.644,0.699)**	3.72	72.95	55.5	67.07
	BAI	0.59(0.562,0.619)	27.17	55.37	60.82	57.2
MHO	BMI	0.512(0.459,0.566)	25.44	84.54	22.3	74.22
	WC	0.59(0.541,0.639)	88.1	45.3	69.59	49.33
	WHtR	0.514(0.465,0.564)	0.58	31.59	74.32	38.68
	VAI	0.568(0.517,0.619)	1.82	68.01	45.95	64.35
	BAI	**0.697(0.651,0.743)**	30.35	69.62	66.89	69.17
MUO	BMI	0.527(0.495,0.559)	26.98	47.25	61.36	53.38
	WC	0.559(0.527,0.59)	91.95	47.25	63.86	54.47
	WHtR	0.531(0.5,0.563)	0.59	40.25	65.83	51.36
	VAI	**0.649(0.619,0.679)**	3.9	67.45	54.2	61.69
	BAI	0.62(0.589,0.651)	29.61	73.35	46.69	61.77

*Abbreviations*: ATP-III, the Adult Treatment Panel-III; MHNO, metabolically healthy non-obese; MUNO, metabolically unhealthy non-obese; MHO, metabolically healthy obese; and MUO, metabolically unhealthy obese; BMI, body mass index; WC, waist circumference; WHtR, waist-to-height ratio; VAI, visceral adiposity index; BAI, body adiposity index.

**Table 3:** Areas under the receiver operating characteristic curve (AUC) for identifying hyperuricemia risk with various adiposity measures within four metabolic health and obesity phenotypes defined by HOMA criteria.

Obesity phenotype		AUC	Threshold	Specificity	Sensitivity	Accuracy
MHNO	BMI	0.553(0.531,0.575)	22.98	73.93	35.32	66.85
	WC	0.611(0.59,0.632)	76.25	47.28	69.43	51.34
	WHtR	0.543(0.521,0.564)	0.47	44.87	64.31	48.44
	VAI	**0.674(0.653,0.696)**	1.63	65.91	61.02	65.01
	BAI	0.614(0.593,0.634)	27	51.49	69.06	54.71
MUNO	BMI	0.593(0.554,0.632)	23.09	67.87	48.25	62.13
	WC	0.624(0.585,0.663)	80.45	55.43	63.99	57.93
	WHtR	0.553(0.514,0.593)	0.51	57.89	55.24	57.11
	VAI	**0.719(0.684,0.755)**	3.7	83.94	48.95	73.69
	BAI	0.579(0.539,0.618)	28.29	45.15	69.93	52.41
MHO	BMI	0.517(0.482,0.552)	26.99	58.32	48.2	55.6
	WC	0.596(0.562,0.629)	89.15	47.7	67.59	53.06
	WHtR	0.514(0.48,0.548)	0.64	7.46	98.34	31.94
	VAI	**0.705(0.672,0.738)**	2.91	76	57.62	71.04
	BAI	0.666(0.633,0.698)	30.67	63.84	65.65	64.33
MUO	BMI	0.553(0.514,0.592)	26.98	46.45	66.18	54.59
	WC	0.575(0.536,0.614)	91.95	45.44	67.34	54.47
	WHtR	0.509(0.469,0.549)	0.56	62.27	42.2	53.99
	VAI	**0.665(0.628,0.703)**	3.02	61.66	65.03	63.05
	BAI	0.62(0.581,0.659)	30.38	72.62	49.13	62.93

*Abbreviations*: HOMA, homeostasis model assessment of insulin resistance; MHNO, metabolically healthy non-obese; MUNO, metabolically unhealthy non-obese; MHO, metabolically healthy obese; and MUO, metabolically unhealthy obese; BMI, body mass index; WC, waist circumference; WHtR, waist-to-height ratio; VAI, visceral adiposity index; BAI, body adiposity index.

### Associations between hyperuricemia, VAI and metabolic health-obesity phenotypes (according to two different criteria)

The VAI was significantly associated with hyperuricemia irrespective of the criteria used to define metabolic health-obesity phenotypes (Table 4). The age- and sex-adjusted ORs (95% CIs) for hyperuricemia were 1.41 (95% CI 1.16–1.73) for the second, 2.29 (95% CI 1.90–2.76) for the third, and 6.93 (95% CI 5.79–8.29) for the fourth VAI quartile when compared with the first VAI quartile (model 1), which demonstrates a strong association between hyperuricemia and VAI. When metabolic obesity phenotypes were included into the model (model 2), this association was attenuated but still highly significant (*P* < 0.001), which demonstrated that VAI was associated with hyperuricemia independent of metabolic obesity phenotype and regardless of criteria. Subjects in the highest VAI quartile had an approximately five-fold increased risk of hyperuricemia compared with their counterparts in the lowest VAI quartile (OR 4.88, 95% CI 3.92–6.09 and OR 5.65, 95% CI 4.68–6.82 according to ATP-III and HOMA-IR criteria, respectively; model 2). This independent association persisted significantly even after adjustment for blood pressure, glucose and other cardiovascular risks (model 3). The adjusted ORs (model 3) of subjects in the highest VAI quartile for hyperuricemia risk were 4.38 (95% CI 3.49–5.49) when metabolic obesity phenotypes were defined by ATP-III criteria, while the adjusted ORs were 5.07 (95% CI 4.18–6.14) when using the HOMA-based criteria. In order to compare the predictive power for hyperuricemia of different obesity indices in logistic regression model, we calculated the probabilities by logistic regression analysis, and then ROC analysis was used to demonstrate the value of the probabilities for hyperuricemia diagnosis, with corresponding AUC values for VAI, BMI and others (Supplementary Figure 2). ROC curve analysis revealed that the largest AUC was observed for VAI (0.813, 95% CI 0.802–0.824 and 0.815, 95% CI 0.804–0.826 for inclusion of ATP-III and HOMA criteria-based obesity phenotypes, respectively), following by WC, WHtR, BMI and BAI. Table 5 shows the association of hyperuricemia with VAI stratified by age groups. Among subjects aged below 65 years, the relations between the three higher VAI quartiles and risk of hyperuricemia were consistently seen in both obesity phenotype definitions; nevertheless, the significance of associations between the 2nd and 3rd quartile of VAI and hyperuricemia disappeared among those aged 65 years and more.

**Table 4:** Adjusted odds ratios (OR) and 95% confidence intervals (CI) of the presence of hyperuricemia associated with the visceral adiposity index, along with metabolic health and obesity phenotypes defined by ATP-III and HOMA criteria, respectively.

	Model 1	ATP-III criteria		HOMA criteria	
		Model 2	Model 3	Model 2	Model 3
**Visceral adiposity index**					
1^st^ Quartile	1 (Ref)	1 (Ref)	1 (Ref)	1 (Ref)	1 (Ref)
2^nd^ Quartile	1.41 (1.16–1.73)	1.36 (1.11–1.66)	1.3 (1.06–1.59)	1.36 (1.11–1.66)	1.29 (1.06–1.58)
3^rd^ Quartile	2.29 (1.90–2.76)	1.95 (1.60–2.38)	1.77 (1.45–2.17)	2.03 (1.67–2.46)	1.85 (1.52–2.24)
4^th^ Quartile	6.93 (5.79–8.29)	4.88 (3.92–6.09)	4.38 (3.49–5.49)	5.65 (4.68–6.82)	5.07 (4.18–6.14)
**Obesity phenotype**					
MHNO	—	1 (Ref)	1 (Ref)	1 (Ref)	1 (Ref)
MUNO		1.29 (1.08–1.54)	1.27 (1.05–1.54)	1.44 (1.20–1.72)	1.54 (1.27–1.87)
MHO		1.19 (0.96–1.48)	1.11 (0.89–1.38)	1.25 (1.06–1.47)	1.16 (0.98–1.38)
MUO		1.93 (1.59–2.35)	1.78 (1.43–2.20)	2.16 (1.79–2.60)	2.07 (1.69–2.53)

*Abbreviations*: ATP-III, the Adult Treatment Panel-III; HOMA, homeostasis model assessment of insulin resistance; MHNO, metabolically healthy non-obese; MUNO, metabolically unhealthy non-obese; MHO, metabolically healthy obese; and MUO, metabolically unhealthy obese. Model 1: Adjusted for age and sex. Model 2: Adjusted for Model 1 + urban/rural resident, smoking status, alcohol status and metabolic health-obesity phenotypes. Model 3: Adjusted for Model 2 + white blood cell, total cholesterol, blood pressure, glucose and hs-CR.

**Table 5:** Adjusted odds ratios (OR) and 95% confidence intervals (CI) of the presence of hyperuricemia associated with the visceral adiposity index, along with metabolic health and obesity phenotypes stratified by age groups.

	ATP-III criteria		HOMA criteria	
	Below 65 years	Above 65 years	Below 65 years	Above 65 years
**Visceral adiposity index**				
1st Quartile	1 (Ref)	1 (Ref)	1 (Ref)	1 (Ref)
2nd Quartile	**1.36 (1.07–1.73)**	1.09 (0.74–1.62)	**1.35 (1.07–1.72)**	1.1 (0.74–1.63)
3rd Quartile	**2.05 (1.62–2.6)**	1.07 (0.71–1.62)	**2.13 (1.69–2.67)**	1.16 (0.78–1.72)
4th Quartile	**4.99 (3.84–6.49)**	**2.78 (1.77–4.39)**	**5.78 (4.61–7.23)**	3.35 (2.25–4.98)
**Obesity phenotype**				
MHNO	1 (Ref)	1 (Ref)	1 (Ref)	1 (Ref)
MUNO	1.29 (1.03–1.61)	1.32 (0.9–1.95)	1.64 (1.31–2.04)	1.24 (0.84–1.85)
MHO	1.1 (0.86–1.41)	1.22 (0.73–2.04)	1.17 (0.97–1.41)	1.21 (0.83–1.77)
MUO	1.71 (1.33–2.18)	2.35 (1.5–3.67)	1.93 (1.53–2.43)	2.71 (1.76–4.18)

*Abbreviations*: ATP-III, the Adult Treatment P anel-III; HOMA, homeostasis model assessment of insulin resistance; MHNO, metabolically healthy non-obese; MUNO, metabolically unhealthy non-obese; MHO, metabolically healthy obese; and MUO, metabolically unhealthy obese. Model was adjusted for age, sex, urban/rural resident, smoking status, alcohol status, metabolic health-obesity phenotypes, white blood cell, total cholesterol, blood pressure, glucose and hs-CR.

Furthermore, when including abdominal obesity into ATP-III definition (the sensitivity analysis), a highly similar pattern was found: the strength of association between VAI, obesity phenotypes and hyperuricemia was almost identical when excluding WC criterion from metabolic abnormality conditions (Supplementary Table 2).

### Association between VAI and hyperuricemia within each stratum of metabolic obesity phenotypes

**Figure 2:**
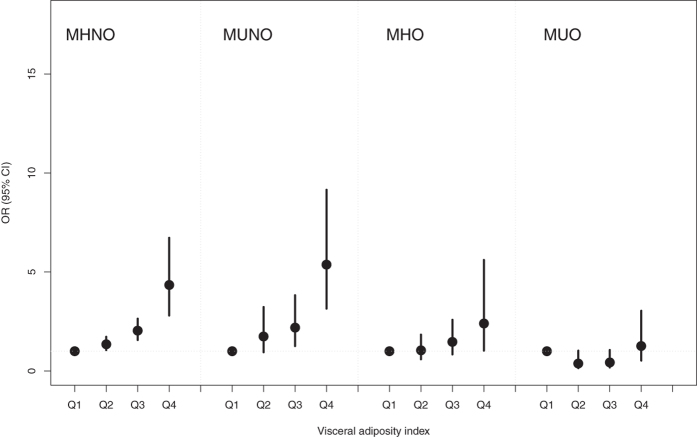
Adjusted odds ratios (OR) and 95% confidence intervals (CI) for hyperuricemia risk associated with visceral obesity index within each metabolic obesity phenotype defined by ATP-III criteria. Vertical bars are 95% CIs. The adjusted OR was obtained from Model 3: adjusted for age, sex, urban/rural resident, smoking status, alcohol status, white blood cell, total cholesterol, blood pressure, glucose and hs-CRP. *Abbreviations*: ATP-III, the Adult Treatment Panel-III; MHNO, metabolically healthy non-obese; MUNO, metabolically unhealthy non-obese; MHO, metabolically healthy obese; and MUO, metabolically unhealthy obese.

**Figure 3:**
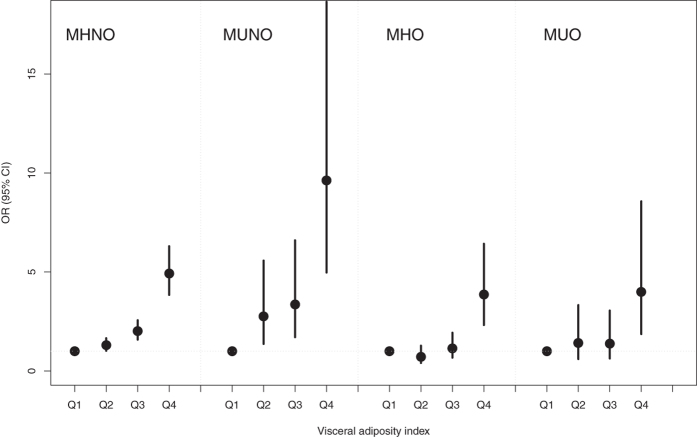
Adjusted odds ratios (OR) and 95% confidence intervals (CI) for hyperuricemia risk associated with visceral obesity index within each metabolic obesity phenotype defined by HOMA criteria. Vertical bars are 95% CIs. The adjusted OR was obtained from Model 3: adjusted for age, sex, urban/rural resident, smoking status, alcohol status, white blood cell, total cholesterol, blood pressure, glucose and hs-CRP. *Abbreviations*: HOMA, homeostasis model assessment of insulin resistance; MHNO, metabolically healthy non-obese; MUNO, metabolically unhealthy non-obese; MHO, metabolically healthy obese; and MUO, metabolically unhealthy obese.

The ORs for hyperuricemia risk as a function of the VAI in an analysis stratified by metabolic health and obesity phenotypes are presented in Figs 2 and 3. The significant association between VAI and risk of hyperuricemia was consistently evident in all except the ATP-III criteria-based MUO phenotype, which indicated again that a significant relationship exists, independent of metabolic health-obesity phenotype. Compared with the lowest quartile of VAI, the OR of hyperuricemia risk in the highest quartile was 5.24 (95% CI 3.42–8.02), 5.62 (95% CI 3.31–9.54) and 2.5 (95% CI 1.13–5.55) in the MHNO, MUNO and MHO phenotypes, respectively when ATP-III criteria were used. Similarly, the adjusted ORs associated with the highest VAI quartile for hyperuricemia using the HOMA criteria were consistently significant, with 9.96- and 3.85-fold increased risks in the MUNO and MUO phenotypes. Again, this is suggestive of a significant relationship that is independent of metabolic health-obesity phenotype. It was also notable that, compared to ATP-III criteria-based metabolic obesity phenotypes, subjects in the 4^th^ quartile of VAI consistently demonstrated higher ORs for hyperuricemia when defined by HOMA criteria.

Next, we sought to investigate the respective effect of obesity status and VAI criteria-based metabolic health status, and their joint effect on OR for hyperuricemia risk (Supplementary Table 4). Compared with participants with a healthy BMI, the participants with obesity (OR = 1.7, 95% CI: 1.5–1.94) had an increased risk of hyperuricemia. As to metabolically unhealthy individuals, they were at a higher risk of developing hyperuricemia (OR = 2.92, 95% CI: 2.56–3.32) when compared with their metabolically healthy counterparts. With respect to different obesity phenotypes, MUNO and MHO subjects had respectively nearly 3.2- and 1.6 fold increased risk of hyperuricemia than their MHNO peers. After further adjustments made for blood pressure and other biomarkers, the association for hyperuricemia were still significant, with corresponding ORs of 2.77 (95% CI: 2.38–3.22) and 1.42 (95% CI: 1.09–1.85) for MUNO and MHO subjects, respectively.

## Discussion

To our knowledge, this is the first large-scale cross-sectional study investigating the relationship between visceral fat, estimated by VAI and risk of hyperuricemia, along with different metabolic health and obesity phenotypes in China. The present study demonstrated that that obesity is an important risk factor of hyperuricemia among a Chinese population, and found the increase of visceral adiposity was positively associated with hyperuricemia risk. Compared to other obesity indices, VAI had a better discriminatory ability in identifying the risk of hyperuricemia than BMI, WC and WHtR. Additionally, our findings highlight that greater VAI was significantly and independently associated with hyperuricemia in models, including two representative definitions of metabolic health and obesity phenotypes. With the current lack of consensus on definition of metabolic health and obesity phenotypes, this consistently significant association, irrespective of the definition for these phenotypes, further emphasizes the impact of visceral adipose tissue on hyperuricemia in Chinese individuals. Furthermore, the risk of hyperuricemia was significantly higher in the highest VAI quartile compared with the reference quartile within each stratum of obesity phenotypes, independent of age, gender, drinking and smoking habits, and other confounders, revealing that VAI is a reliable indicator of the presence of hyperuricemia in adults.

Recently, many studies have reported a significant relationship between obesity and hyperuricemia^50, 51^, but very few have focused on the association of visceral adiposity with hyperuricemia. In an early study of only 50 apparently healthy Japanese men aged 29–78 years, Takahashi *et al*. found that visceral adiposity, estimated by visceral fat area (VFA), was positively correlated with UA metabolism and contributed more to increased UA levels than BMI, which suggests there is an adverse effect of visceral adiposity on UA^52^. Consistent with this, in a small study of only 15 healthy and 31 obese men, Matsuura *et al*. demonstrated that serum UA levels were higher in both the subcutaneous obesity and the visceral obesity groups than in the non-obese control group^21^. Based on another Japanese cross-sectional study of 508 male workers aged 24–68 years, the investigators showed a significant correlation between CT-measured VFA and UA, as well as a close association of increased visceral adiposity and risk of high UA levels, independently of BMI, BP and other confounders^53^. Similar findings were observed in a cross-sectional study of 699 Korean subjects with diabetes^54^. In this cohort, using CT-measured VFA, visceral adiposity was positively associated with serum UA levels, and more importantly, subjects with high visceral adiposity tertile had a 2.33-fold increased risk of hyperuricemia after adjustment for age, sex and other potential confounders^54^. However, this is in partial agreement with Matsuura *et al*. and Hikita *et al*. studies in that significance was only found in the association between VFA and serum UA levels/hyperuricemia, and was absent when using subcutaneous fat area as an estimate of visceral adiposity. The reason for this discrepancy could be due to specific study designs. Only individuals with diabetes were included in Kim *et al*.’s study^54^, and they had altered distributions of abdominal adipose tissue compared with subjects with normal glucose tolerance^55^. Finally, a very recent cross-sectional study of 801 men aged 28–76 years, performed in Japan by Yamada *et al*. and using estimates of CT-measured intra-abdominal fat area, found a significant association between visceral adiposity and hyperuricemia. The adjusted OR in the highest quintile of visceral adiposity was 4.72 after adjustment for age, lifestyles and other risk factors^30^. In the present study, our results were in line with previous findings, with a more than four-fold increased risk of hyperuricemia observed in subjects with high visceral adiposity levels. Moreover, this significant association was independent of age, sex and, especially, metabolic health and obesity phenotype irrespective of its definitions. All this evidence suggests that visceral adiposity is an important risk factor for hyperuricemia.

Currently, visceral adiposity assessment is suggested in many situations and is becoming routine clinical practice. Although imaging techniques such as CT and MRI provide the most accurate measurements of visceral adiposity, they are seldom available in daily practice since they are expensive, may involve exposure to radiation, and have practical and ethical issues. Alternatively, on the basis of recent investigations, the potential use of VAI as a surrogate marker of visceral adiposity should be highlighted. The VAI, which relies on both anthropometric and metabolic parameters, was first proposed by Amato *et al*. for estimating visceral adiposity dysfunction associated with cardiometabolic risk^33^. As indicated in their subsequent study using a relatively large Italian cross-sectional sample, the authors suggested age-stratified cut-off points for VAI, which were capable of providing information regarding visceral adiposity tissue function^34^. Therefore, the VAI has been considered to be a useful surrogate for visceral adiposity tissue measured by MRI^33^. This finding was further justified in a study conducted in a Korean cohort, which confirmed the significance of VAI as a marker for CT-measured visceral adiposity, and highlighted its generalizability to various ethnic groups^56^. Since then, investigations of association between VAI and different health-related outcomes have recently appeared. In a Chinese community-based survey including 4065 subjects (mean aged 45 years), subjects with the highest VAI quartile were shown to have more than 1.5-fold increased risk of hypertension irrespective of gender, compared to their counterparts with the lowest VAI quartile^37^. Similarly, in another Chinese population-based cohort of 2142 subjects aged 18–85 years, the investigators found that women in the upper quartiles of VAI scores had approximately twice the risk of chronic kidney disease, after controlling for age and other potential confounders, compared to those in the lowest quartile^57^. This positive association was relatively attenuated but still significant after accounting for diabetes and hypertension, especially in female subjects. These compelling results recognize the importance of VAI for identifying various diseases, and were confirmed in a longitudinal study^58^. During more than 5-years follow-up of 3461 Chinese people aged 35–74 years, Chen *et al*. found that, compared to BMI, WC and WHtR, VAI had superior capacity to discriminate for diabetes, with the highest AUC value being 0.62^58^. Moreover, subjects with the highest VAI scores carried a 2.55-fold increased risk of diabetes compared with their counterparts with the lowest VAI scores. Another recent study investigating both diabetes and pre-diabetes has also reached similar conclusions^39^. However, a Tehran-based 6-year follow-up study of 5964 subjects reported that VAI could be used as a screening tool for identifying the risk of diabetes, but is unlikely to be superior to WHtR^38^. This discrepancy might be attributed to different characteristics of the study populations, the duration of follow-ups, and the numbers of events between the two studies. In the present study, the risk of hyperuricemia in the highest VAI group was five times greater than in the lowest VAI group in non-obese subjects, and 2.5 times greater in MHO subjects. Our findings support the hypothesis that VAI is a convenient prognostic tool for predicting hyperuricemia, especially within different metabolic health and obesity phenotypes.

Visceral obesity is thought to be directly linked to deterioration of insulin sensitivity, increased risk of developing diabetes, and more specifically, “high-TG/low HDL-C dyslipidemia”^33^. For instance, Rothney *et al*. and Sasai *et al*. both showed that visceral adiposity, measured by Dual-Energy X-ray Absorptiometry (DXA), was significantly associated with HDL-C and TG after controlling for age and other confounders^59, 60^. Moreover, 1-kg increase in visceral adiposity corresponds to a 0.13 mmol/l (5.1 mg/dL) decrease in HDL-C and 0.29 mmol/l (26.1 mg/dL) increase in TG^60^. As the proxy of visceral adiposity, we speculated that the VAI could also be used as a proxy of dyslipidemia due to inclusion of HDL-C and TG in its formulas. Indeed, based on a cross-sectional study of 221 elderly in Brazil, the investigators found that the VAI had the strongest correlation with FPG, TG, TC, HDL-C and BP, and more importantly, the VAI was good predictor of hypertriglyceridemia (OR 3.64, 95% CI 2.48–5.35) and reduced HDL-C (OR 2.26, 95% CI 1.81–2.83)^61^. Similarly, Knowles *et al*. in a cross-sectional study with 1518 Peruvian adults highlighted a significant association of VAI with all the components of metabolic syndrome, especially for hypertriglyceridemia and low HDL-C in both genders^62^. In coincidence with the previous findings, Schuster *et al*., from a cross-sectional study including 444 young Brazilians, demonstrated that VAI strongly correlated to FPG, HDL-C, and TG, with a higher AUC for increased TG and low HDL-C, irrespective of gender^63^. They also showed a positive association between VAI and risk of hypertriglyceridemia and (OR 30.74) and low HDL-C (OR 3.95), adding again to the evidence that VAI could be a proxy of dyslipidemia. Furthermore, it is noteworthy mentioning another small study including only 24 patients with craniopharyngioma in Italy. After a follow-up of 15 years, Ferrau *et al*. found that increased VAI significantly correlated with the occurrence of metabolic syndrome, TG, HDL-C and HOMA-IR, and these associations were not influenced by gender and age of disease onset^64^. Taken together, these findings confirmed a strong relationship between VAI and dyslipidemia, and considering an inclusion of both HDL-C and TG in the VAI formula, this relationship appears expected; therefore, VAI could serve as a proxy of dyslipidemia.

Various studies have demonstrated MUNO and MUO subjects to have higher risks of hyperuricemia^65^, hypertension^66^, diabetes^67–69^, CVD^70^ and mortality^25, 71^, in both Asian and Western populations. However, there is still controversy over whether subjects within the MHO group carry an increased risk of diseases compared with the MHNO phenotype. In a cohort study of 7122 British people aged 39–63 years after a follow up of 17 years, Hinnouho *et al*. concluded that MHO individuals had an increased risk of incident CVD and diabetes, with adjusted hazard ratios of 1.97 and 3.25, compared with their healthy counterparts, regardless of the metabolic health definition used, except for HOMA^67^. Similarly, in a prospective cohort of 2352 community-dwelling Korean adults followed for 8 years, Lee *et al*. reported that the MHO group had a 2.2-fold increased risk of developing hypertension^72^. This study stressed that the status of MHO does not appear to have a protective effect against the development of hypertension. This finding was confirmed in recent individual participant data meta-analyses, including two 10-year follow-up study^73^, adding to the evidence that MHO subjects have an elevated risk of hypertension, rather than having a benign conditions. Moreover, in a prospective cohort of 31834 Korean men followed-up after 5 years, MHO subjects were nearly 1.5 times more likely to develop diabetes than healthy counterparts^68^. Consistent with these studies, the determining role of obesity on hyperuricemia in MHO phenotypes was observed in a large cross-sectional study conducted by Chen *et al*.^65^. In this cohort including 11435 Chinese rural people aged ≥35 years, the authors investigated the association of the MHO phenotype with hyperuricemia risk^65^. They found that increased weight resulted in a higher prevalence of hyperuricemia, and the latter ranged 17.9–29% and 7–14% for MHO and MUO men and women, respectively. Moreover, when compared with MHNO subjects, individuals with MHO and MUO were 2.48- and 4.81-fold more likely to develop hyperuricemia, even after adjusting for age, sex and other potential confounders. In contrast, some studies have concluded that MHO individuals are not at a significantly increased risk of diabetes^69, 74^, stroke^69^ and CVD^75, 76^. Even a protective effect of MHO status has been reported^25^. Indeed, Yang *et al*., in a nationwide population-based cohort study with a 96-month follow-up, found a significantly lower risk of cancer, cardiovascular and all-cause mortality within the MHO phenotype, compared to their healthy counterparts^25^, which appears to show that MHO is beneficial for health-related outcomes. In this study, when defining metabolic abnormality using the HOMA criterion, our results showed that the MHO phenotype was significantly associated with a higher hyperuricemia risk that was independent of age, sex and other confounders; however, this was not the case when using the ATP-III criterion. Even though the precise reasons for the discrepancy were unable to be ascertained, differences in definitions of metabolic abnormality could be a relevant factor.

The most commonly proposed mechanisms underlying visceral adiposity to hyperuricemia are: excessive UA production, and a reduction in the extrarenal excretion of UA related to visceral fat accumulation, or even a combination of the two^30, 77^. As demonstrated by several studies, pathologic visceral adipose tissue has been considered to be metabolically active. It enables the regulation of substantial amounts of adipocytokines, resulting in conditions of adipocytokine dysregulation such as hyperinsulinemia^78–80^. Since hyperinsulinemia is believed to increase the reabsorption of sodium and UA on renal tubules, thereby reducing urinary excretion and urinary sodium excretion, it consequently causes hyperuricemia^81, 82^. It is worthwhile mentioning another important study that used bidirectional Mendelian randomization approach to explore the nature and direction of causality between serum UA and adiposity^83^. Based on a population-based study of Caucasians aged 35–75 years, Lyngdoh *et al*. found that serum UA (explained by a proxy of a gene instrument the SLC2A9) was not associated to fat mass. In contrast, adiposity makers explained by genetic variants were positively and significantly associated with serum UA^83^. Therefore, the authors suggested that elevated serum UA is a consequence rather than a cause of adiposity. Moreover, this result is compatible with the hypothesis that hyperinsulinemia, a consequence of visceral adiposity accumulation, enhances renal proximal tubular reabsorption of UA with a subsequent increase in serum UA levels. Lyngdoh *et al*.’s evidence for this causality can be considered to be sufficiently strong, because genetic variants are not influenced by any confounders. In our study, the metabolically healthy status phenotypes defined by insulin resistance (HOMA-IR) were significantly associated with hyperuricemia, which supports the link to hyperinsulinemia.

Another plausible mechanism for this link, as suggested by numerous investigators, is the strong association of visceral fat accumulation with UA overproduction^21, 54, 84^. Increased visceral fat accumulation provides over-flow of free fatty acids to the liver through the portal vein. It has been confirmed that excess free fatty acids - products of lipolysis - may enhance TG synthesis in the liver, leading to hypertriglyceridemia^21^. Also by means of the pentose phosphate pathway, an excessive inflow of free fatty acids may be linked to de novo purine synthesis, which is responsible for accelerating the production of UA^85^. This explanation appears to be supported by previous studies that have found a significant association between TG and UA. Indeed, Matsuura *et al*. observed a significant positive correlation between TG and serum UA levels, and more importantly, visceral fat-type obesity was significantly related to excessive UA production^21^. Consistent with this, Kim *et al*. found that visceral fat area (a surrogate of visceral adiposity), was closely correlated with serum TG levels, which in turn were positively associated with serum UA levels, after adjustment for age, sex and other confounders^54^. This is further evidence of a relationship between UA production and TG synthesis. In the present study, TG levels were positively associated with the UA levels after adjusting for age, sex, lifestyle, BMI, BP, serum creatinine, haemoglobin, HDL-C, FBG and HbA1c (*b*-coefficient = 29.70, *P* < 0.001; data not shown). Taken together, all these pieces of evidence indicate that there is a relationship between UA production and triglyceride synthesis.

Several limitations of the present study should be acknowledged. First, due to the nature of the cross-sectional design, the findings of this study do not explicitly infer a causal relationship between the VAI and hyperuricemia risk. Thus, we must be cautious in interpreting the present results, and further cohort studies are warranted to clarify our findings. Second, the VAI was first developed in Caucasian populations, and its suitability as surrogate of visceral fat for study relationship with various health outcomes in other populations needs to be further investigated. However, one early study has already validated the use of the VAI for Chinese individuals^36^, and our research revealed that a significant relationship between VAI and hyperuricemia. There still remains much work to do in developing a well-designed study that determines the mechanism underlying this relationship. Finally, consensus on the definitions of metabolic obesity phenotypes is currently lacking. Therefore, observed associations between obesity phenotypes and health-related outcomes can vary according to the definition used. In our study, VAI scores were significantly and positively associated with hyperuricemia risk, regardless of the obesity phenotype definition used, which suggests that our findings were consistent.

However, despite these limitations, the notable strengths and contributions of this study should be highlighted. First of all, although the cross-sectional nature of our study was not an optimal design, we were able to demonstrate that the VAI is a suitable predictor of hyperuricemia risk. This compelling result validates a close association of the VAI with hyperuricemia risk that is independent of the various obesity phenotypes. Therefore, the longitudinal relationship between the VAI and hyperuricemia incidence warrants investigation. Second, with its large sample size, the present study has reasonable statistical power that likely reflects the real associations. In addition, the study sample came from a nationally representative survey, which should minimize the possibility of sample selection bias. Third, anthropometric measurements and serum analysis were obtained by trained study personnel following a standard protocol, which should rule out the effect of measurement bias. Finally, to minimize the potential for confounding, we adjusted our models for several covariates, including age, sex, alcohol consumption, smoking and other serum measurements.

In conclusion, by using a nationwide, population-based Chinese cohort, our study provides compelling evidence that the VAI is a simple predictor of hyperuricemia that is superior to BMI, WC or WHtR measurements. The association of the VAI with hyperuricemia was significant, especially if metabolic health and obesity phenotypes were concomitantly present, and was independent of their definitions. In large-scale studies, the VAI could be used as a convenient surrogate for visceral adiposity measurements, enabling the efficient assessment of various health risks in the Chinese population.

## Electronic supplementary material

Supplementary Material
