# Supplementary material for: Visceral adiposity index is strongly associated with hyperuricemia independently of metabolic health and obesity phenotypes
Source: Sci Rep. 2017 Aug 18;7:8822. doi: 10.1038/s41598-017-09455-z (PMC5562916; doi:10.1038/s41598-017-09455-z)

**Title:** Visceral adiposity index is strongly associated with hyperuricemia independently of metabolic health and obesity phenotypes

**Author** : Huimin Dong^1^, Yang Xu^2^,Xiuzhi Zhang^2^, Simiao Tian^2^

**^1^**Department of Clinical Nutrition and Metabolism, Affiliated Zhongshan Hospital of Dalian University, NO. 6 Jiefang Street Zhongshan District, Dalian, Liaoning Province, People’s Republic of China, 116001

**^2^**Department of Scientific Research Project, Affiliated Zhongshan Hospital of Dalian University, NO. 6 Jiefang Street Zhongshan District, Dalian, Liaoning Province, People’s Republic of China, 116001

**Corresponding author**: Simiao Tian

Address correspondence to Dr. Simiao Tian at Department of Scientific Research Project, Affiliated Zhongshan Hospital of Dalian University, NO. 6 Jiefang Street Zhongshan District, Dalian, Liaoning Province, People’s Republic of China, 116001. Telephone: +86 (0)411 62887084. Fax: +86 (0)411 62893555. E-mail: Simiao_Tian@sina.com

Supplementary Table 1. Areas under the receiver operating characteristic curve (AUC) for identifying hyperuricemia risk with various adiposity measures within four metabolic health and obesity phenotypes defined by ATP-III criteria (including abdominal obesity: waist circumference cut-off is 85 cm for men and 80 cm for women, respectively).

| Obesity phenotype |  | AUC | Threshold | Specificity | Sensitivity | Accuracy |
| --- | --- | --- | --- | --- | --- | --- |
| MHNO | BMI | 0.492(0.468,0.517) | 16.8 | 98.03 | 3.07 | 83.46 |
|  | WC | 0.567(0.543,0.59) | 76.25 | 52.36 | 57.98 | 53.22 |
|  | WHtR | 0.499(0.475,0.523) | 0.51 | 75.32 | 26.84 | 67.88 |
|  | VAI | 0.63(0.606,0.654) | 1.5 | 66.45 | 54.29 | 64.59 |
|  | BAI | **0.637(0.615,0.66)** | 26.69 | 53.42 | 69.94 | 55.95 |
| MUNO | BMI | 0.544(0.511,0.578) | 22.98 | 48.93 | 59.12 | 52.78 |
|  | WC | 0.549(0.516,0.583) | 84.05 | 51.47 | 57.8 | 53.87 |
|  | WHtR | 0.536(0.502,0.569) | 0.52 | 50.27 | 58.9 | 53.53 |
|  | VAI | **0.668(0.636,0.7)** | 3.72 | 67.51 | 60.66 | 64.92 |
|  | BAI | 0.599(0.566,0.632) | 27.52 | 54.68 | 62.64 | 57.69 |
| MHO | BMI | 0.517(0.465,0.569) | 25.44 | 84.24 | 22.88 | 74.05 |
|  | WC | 0.584(0.535,0.633) | 88.1 | 47.01 | 67.32 | 50.38 |
|  | WHtR | 0.517(0.467,0.566) | 0.58 | 30.6 | 75.16 | 38 |
|  | VAI | 0.57(0.52,0.621) | 1.82 | 66.54 | 47.71 | 63.41 |
|  | BAI | **0.7(0.655,0.745)** | 30.35 | 69.4 | 67.97 | 69.16 |
| MUO | BMI | 0.523(0.491,0.555) | 26.98 | 46.31 | 61.91 | 53.18 |
|  | WC | 0.549(0.517,0.581) | 91.95 | 45.45 | 64.44 | 53.82 |
|  | WHtR | 0.542(0.51,0.575) | 0.54 | 78.55 | 28.7 | 56.6 |
|  | VAI | **0.645(0.614,0.675)** | 3.9 | 66.9 | 54.15 | 61.29 |
|  | BAI | 0.621(0.589,0.652) | 30.67 | 64.49 | 55.42 | 60.49 |

*Abbreviations*: ATP-III, the Adult Treatment Panel-III; MHNO, metabolically healthy non-obese; MUNO, metabolically unhealthy non-obese; MHO, metabolically healthy obese; and MUO, metabolically unhealthy obese; BMI, body mass index; WC, waist circumference; WHtR, waist-to-height ratio; VAI, visceral adiposity index; BAI, body adiposity index.

Supplementary Table 2. Adjusted odds ratios (OR) and 95% confidence intervals (CI) of the presence of hyperuricemia associated with the visceral adiposity index, along with metabolic health and obesity phenotypes defined by ATP-III criteria including abdominal obesity.

|  |  | ATP-III criteria | |
| --- | --- | --- | --- |
|  | Model 1 | Model 2 | Model 3 |
| **Visceral adiposity index** |  |  |  |
| 1^st^ Quartile | 1 (Ref) | 1 (Ref) | 1 (Ref) |
| 2^nd^ Quartile | 1.41 (1.16-1.73) | 1.36 (1.12-1.67) | 1.31 (1.07-1.6) |
| 3^rd^ Quartile | 2.29 (1.90-2.76) | 1.96 (1.61-2.39) | 1.8 (1.47-2.2) |
| 4^th^ Quartile | 6.93 (5.79-8.29) | 4.84 (3.9-6.02) | 4.41 (3.54-5.51) |
| **Obesity phenotypes** |  |  |  |
| MHNO | -- | 1 (Ref) | 1 (Ref) |
| MUNO |  | 1.33 (1.11-1.61) | 1.28 (1.05-1.56) |
| MHO |  | 1.15 (0.94-1.42) | 1.06 (0.86-1.32) |
| MUO |  | 1.94 (1.62-2.34) | 1.76 (1.44-2.16) |

*Abbreviations*: ATP-III, the Adult Treatment Panel-III; MHNO, metabolically healthy non-obese; MUNO, metabolically unhealthy non-obese; MHO, metabolically healthy obese; and MUO, metabolically unhealthy obese. Model 1: Adjusted for age and sex. Model 2: Adjusted for Model 1+ urban/rural resident, smoking status, alcohol status and metabolic health-obesity phenotypes. Model 3: Adjusted for Model 2+ white blood cell, total cholesterol, blood pressure, glucose and hs-CR.

Supplementary Table 3. Characteristics of subjects according to obesity status (defined by body mass index) and metabolic health status defined by VAI criteria.

|  | Metabolically healthy  (MHNO)  (n=3005) | Metabolically unhealthy  (MUNO)  (n=2448) | Metabolically healthy  (MHO)  (n=589) | Metabolically unhealthy  (MUO)  (n=1590) |
| --- | --- | --- | --- | --- |
| Age, year | 47.4 (36.9-59.8) | 51.7 (40.2-61.7) | 50.1 (41.6-60) | 52.4 (43.1-61.2) |
| Sex (female), n (%) | 1733 (57.7%) | 1200 (49%) | 380 (64.5%) | 818 (51.4%) |
| Smoker, n (%) | 866 (28.8%) | 879 (35.9%) | 114 (19.4%) | 476 (29.9%) |
| Alcohol drinker, n (%) | 950 (31.6%) | 804 (32.8%) | 165 (28%) | 553 (34.8%) |
| Urban resident, n (%) | 2104 (70%) | 1644 (67.2%) | 400 (67.9%) | 1069 (67.2%) |
| BMI, kg/m^2^ | 21.2 (19.6-22.7) | 22.5 (20.9-23.7) | 26.5 (25.8-28) | 27 (25.9-28.7) |
| WC, cm | 76 (70.5-81) | 81 (76-87) | 89 (84-94) | 92.5 (87-98) |
| WHtR | 0.5 (0.4-0.5) | 0.5 (0.5-0.5) | 0.6 (0.5-0.6) | 0.6 (0.5-0.6) |
| VAI | 1 (0.7-1.2) | 2.6 (2-3.8) | 1.1 (0.9-1.4) | 3.1 (2.2-4.8) |
| BAI | 26.7 (24.2-29.4) | 27.1 (24.5-29.7) | 31.9 (29.2-34.7) | 31.3 (28.3-34.5) |
| HDL-C, mmol/l | 1.6 (1.4-1.8) | 1.2 (1.1-1.4) | 1.5 (1.4-1.7) | 1.2 (1-1.4) |
| LDL-C, mmol/l | 2.7 (2.2-3.3) | 3 (2.3-3.6) | 3 (2.5-3.6) | 3.1 (2.6-3.8) |
| DBP, mm Hg | 78 (70-82) | 80 (71.7-86) | 80.7 (76.7-90) | 82.7 (79.3-90.7) |
| SBP, mm Hg | 118.7 (109.3-128.7) | 120 (110.7-132) | 124.7 (118-139.3) | 128.7 (119.3-140.7) |
| FPG, mmol/l | 4.9 (4.6-5.3) | 5.1 (4.7-5.6) | 5.1 (4.7-5.5) | 5.3 (4.9-5.9) |
| TC, mmol/l | 4.5 (4-5.1) | 4.8 (4.2-5.5) | 4.8 (4.2-5.4) | 5 (4.5-5.7) |
| TG, mmol/l | 0.8 (0.6-1) | 1.7 (1.4-2.4) | 0.9 (0.7-1.1) | 2 (1.5-2.7) |
| Uric acid, mmol/l | 261 (214-316) | 311 (252-374) | 263 (217-323) | 333 (279-400) |
| HOMA-IR | 1.9 (1.3-2.6) | 2.5 (1.7-3.7) | 2.4 (1.7-3.4) | 3.3 (2.3-5.3) |
| hsCRP | 1 (0-2) | 1 (1-2) | 1 (1-2) | 2 (1-4) |
| HbA1c, % | 5.4 (5.1-5.7) | 5.5 (5.2-5.8) | 5.6 (5.2-5.9) | 5.7 (5.4-6) |
| Diabetes, n (%) | 53 (1.8%) | 135 (5.5%) | 16 (2.7%) | 128 (8.1%) |
| Dyslipidemia, n (%) | 233 (7.8%) | 1154 (47.1%) | 76 (12.9%) | 952 (59.9%) |
| Hypertension, n (%) | 580 (19.3%) | 678 (27.7%) | 216 (36.7%) | 697 (43.8%) |

Data are n (%) or median (interquartile range).

Abbreviations: ATP-III, the Adult Treatment Panel-III; BMI, body mass index; WC, waist circumference; WHtR, waist-to-height ratio; VAI, visceral adiposity index; BAI, body adiposity index; HDL-C, high-density lipoprotein cholesterol; LDL-C, low-density lipoprotein cholesterol; DBP, diastolic blood pressure; SBP, systolic blood pressure; FPG, fasting plasma glucose; TC, total cholesterol; TG, triglycerides; HOMA-IR, homoeostatic model assessment of insulin resistance; hsCRP, high-sensitivity C-reactive protein.

Supplementary Table 4. The odds ratios of hyperuricemia by body mass index categories, VAI criteria-based metabolic health status and cross-classification of BMI-VAI (The covariate “VAI” was not included in the model).

|  | Model 1 | Model 2 |
| --- | --- | --- |
| **Obesity (BMI cut-off 25 kg/m^2^)** |  |  |
| Non-obese | 1 (Ref) | 1 (Ref) |
| obese | 2.09 (1.85-2.36) | 1.7 (1.5-1.94) |
|  |  |  |
| **Metabolic status (defined using VAI criteria)** |  |  |
| Healthy | 1 (Ref) | 1 (Ref) |
| Unhealthy | 3.43 (3.03-3.89) | 2.92 (2.56-3.32) |
|  |  |  |
| **Metabolic health and obesity phenotypes** |  |  |
| MHNO | 1 (Ref) | 1 (Ref) |
| MUNO | 3.12 (2.69-3.61) | 2.77 (2.38-3.22) |
| MHO | 1.62 (1.25-2.11) | 1.42 (1.09-1.85) |
| MUO | 4.88 (4.16-5.73) | 3.83 (3.23-4.54) |

*Abbreviations*: BMI, body mass index; VAI, visceral adiposity index; MHNO, metabolically healthy non-obese; MUNO, metabolically unhealthy non-obese; MHO, metabolically healthy obese; and MUO, metabolically unhealthy obese. Model 1: Adjusted for age, sex, urban/rural resident, smoking status, alcohol status and metabolic health-obesity phenotypes. Model 2: Adjusted for Model 1+ white blood cell, total cholesterol, blood pressure, glucose and hs-CR.

Supplementary Figure 1. Prevalence of hyperuricemia according to metabolic health and obesity status (metabolic obesity phenotypes). The metabolic health status was defined by ATP-III, HOMA and VAI criteria, respectively; the obesity status was defined by body mass index.

*Abbreviations*: ATP-III, the Adult Treatment Panel-III; HOMA, homeostasis model assessment of insulin resistance; VAI, visceral adiposity index; MHNO, metabolically healthy non-obese; MUNO, metabolically unhealthy non-obese; MHO, metabolically healthy obese; and MUO, metabolically unhealthy obese.


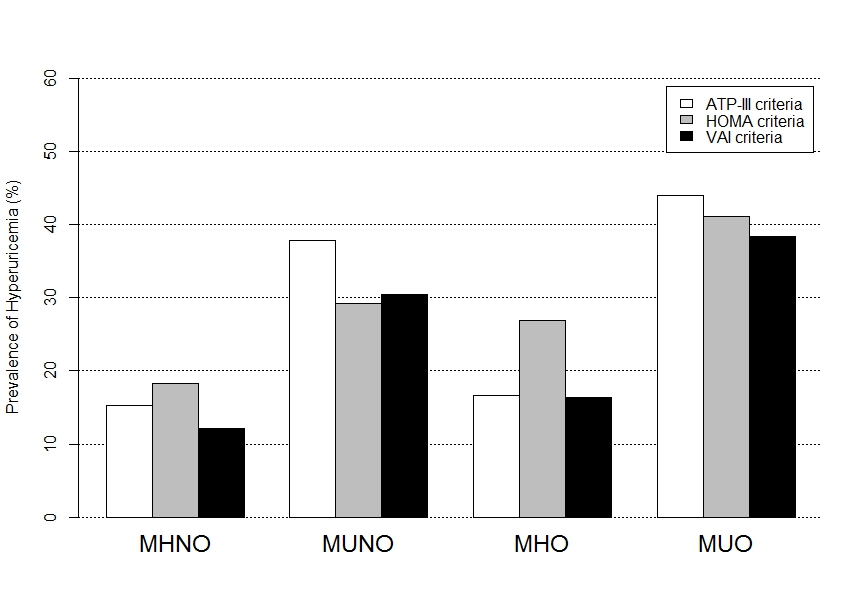


Supplementary Figure 2. Receiver-operating characteristic (ROC) curves of different obesity indices to predict subjects with hyperuricemia. (a) ATP-III criteria-based obesity phenotypes were included in the model, and (b) HOMA criteria-based obesity phenotypes were included in the model. BMI, body mass index; WC, waist circumference; WHtR, waist-to-height ratio; VAI, visceral adiposity index; BAI, body adiposity index.


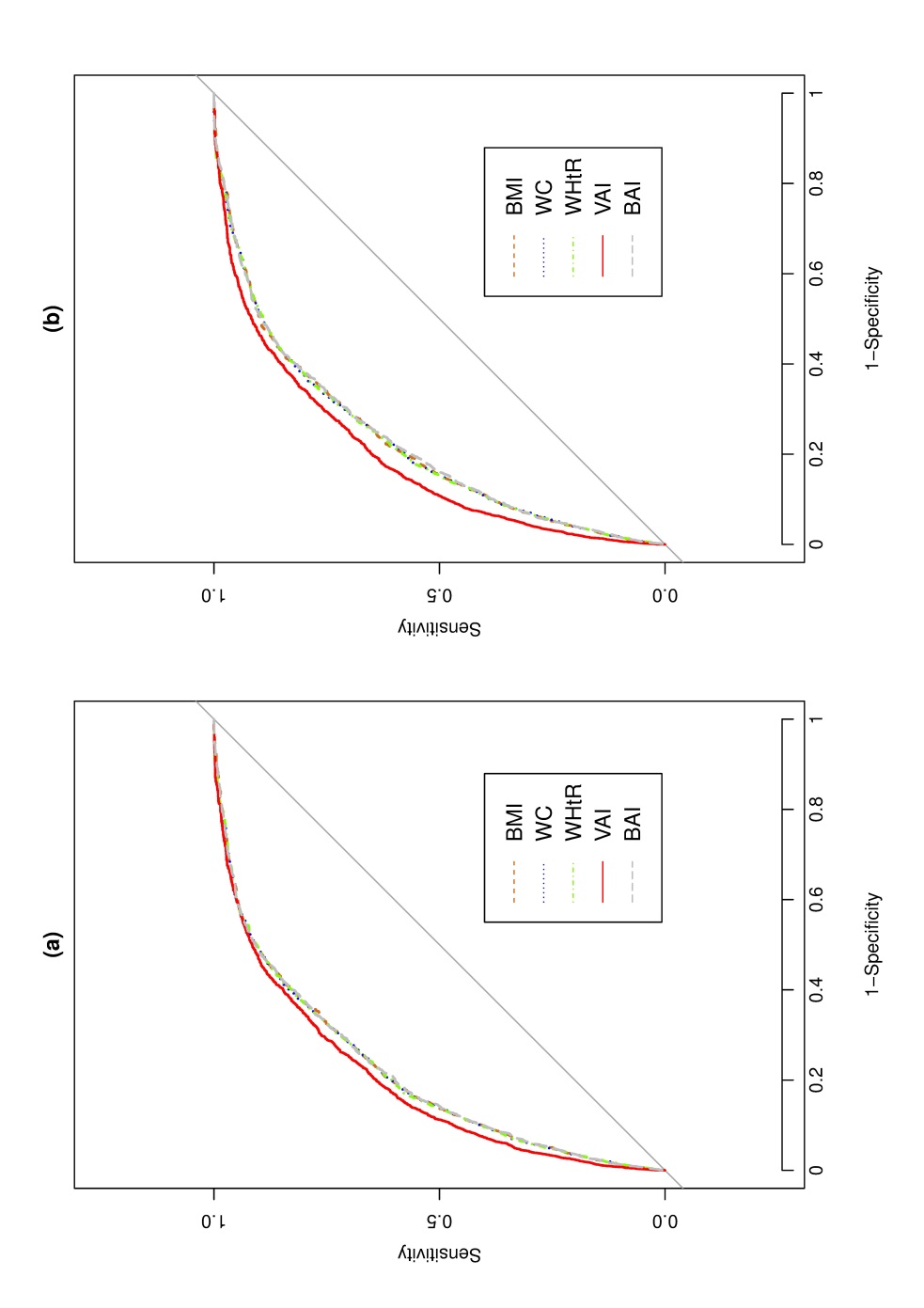

Supplement: Supplementary file 1 — Supplementary Material [file 41598_2017_9455_MOESM1_ESM.doc]
